# DPP8 is a novel therapeutic target for multiple myeloma

**DOI:** 10.1038/s41598-019-54695-w

**Published:** 2019-12-02

**Authors:** Tsutomu Sato, Ayumi Tatekoshi, Kohichi Takada, Satoshi Iyama, Yusuke Kamihara, Paras Jawaid, Mati Ur Rehman, Kyo Noguchi, Takashi Kondo, Sayaka Kajikawa, Kotaro Arita, Akinori Wada, Jun Murakami, Miho Arai, Ichiro Yasuda, Nam H. Dang, Ryo Hatano, Noriaki Iwao, Kei Ohnuma, Chikao Morimoto

**Affiliations:** 1 Department of Hematology, Toyama University Hospital, Toyama, Japan; 2 Department of Medical Oncology and Hematology, Sapporo Medical University, Sapporo, Japan; 3 Department of Hematology, National Cancer Center Hospital East, Kashiwa, Japan; 4 Department of Radiology, University of Toyama, Toyama, Japan; 5 Department of Pediatrics, University of Toyama, Toyama, Japan; 6 Department of Gastroenterology and Hematology, University of Toyama, Toyama, Japan; 7 Division of Hematology/Oncology, University of Florida, Gainesville, Florida USA; 8 Department of Therapy Development and Innovation for Immune Disorders and Cancers, Juntendo University, Tokyo, Japan

**Keywords:** Myeloma, Apoptosis

## Abstract

Dipeptidyl peptidases (DPPs) are proteolytic enzymes that are ideal therapeutic targets in human diseases. Indeed, DPP4 inhibitors are widely used in clinical practice as anti-diabetic agents. In this paper, we show that DPP4 inhibitors also induced cell death in multiple human myeloma cells. Among five DPP4 inhibitors, only two of them, vildagliptin and saxagliptin, exhibited apparent cytotoxic effects on myeloma cell lines, without any difference in suppression of DPP4 activity. As these two DPP4 inhibitors are known to have off-target effects against DPP8/9, we employed the specific DPP8/9 inhibitor 1G244. 1G244 demonstrated anti-myeloma effects on several cell lines and CD138+ cells from patients as well as in murine xenograft model. Through siRNA silencing approach, we further confirmed that DPP8 but not DPP9 is a key molecule in inducing cell death induced by DPP8/9 inhibition. In fact, the expression of DPP8 in CD38+ cells from myeloma patients was higher than that of healthy volunteers. DPP8/9 inhibition induced apoptosis, as evidenced by activated form of PARP, caspases-3 and was suppressed by the pan-caspase inhibitor Z-VAD-FMK. Taken together, these results indicate that DPP8 is a novel therapeutic target for myeloma treatment.

## Introduction

Dipeptidyl peptidases (DPPs) are members of the serine protease subfamily S9B including DPP4, DPP8, DPP9 and fibroblast activation protein (FAP). DPPs selectively cleave N-terminal dipeptides (Xaa-Pro) from their substrates; therefore, they are considered to be ideal drug targets for the treatment of human diseases^1–6^. Notably, DPP4, also known as CD26, cleaves and inactivates the incretins such as glucagon-like peptide-1 (GLP-1) and glucose-dependent insulinotropic peptide (GIP)^7^. These proteins are secreted from enteroendocrine K and L cells and stimulate pancreatic beta cells so as to secrete insulin. An increased GLP-1 level is one of the major effects of DPP4-inhibitor treatment. DPP4 has thus become a molecular target for the management of diabetes mellitus (type 2)^8^. DPP4 inhibitors are now commonly used in clinical practice as anti-diabetic drugs to obtain satisfactory glycemic control in patients with type 2 diabetes mellitus^9^.

Meanwhile, DPP4/CD26 is expressed on various cells and has a multitude of biological functions^10^. Numerous previous reports have demonstrated that CD26 is involved in T-cell function and regulation of the immune system^11–19^. Moreover, DPP4/CD26 is detectable on many types of cancer cells; examples include thyroid carcinoma, gastrointestinal stromal tumor, prostate carcinoma, lung carcinoma, hepatic cancer, colon carcinoma, renal cell cancer (RCC), and malignant pleural mesothelioma (MPM). Hematologic cancers such as T-acute lymphoblastic leukemia, T-anaplastic large cell lymphoma, and T-lymphoblastic lymphoma are also included^20–22^.

We previously reported that DPP4/CD26 is not expressed on normal mesothelial cells but on MPM cells^23^, therefore, CD26 is a potential therapeutic target in the management of MPM patients^24^. In addition, our *in vivo* experiments confirm the anti-tumor effects of anti-CD26 monoclonal antibody in murine xenograft systems of MPM^25–27^ or RCC^28^. Expanding on our preclinical findings, we reported the promising results of the first-in-human phase 1 clinical study of YS110, an anti-CD26 recombinant DNA-derived humanized monoclonal antibody, regarding pharmacokinetics, pharmacodynamics, safety, and preliminary anti-tumor activities in patients with refractory MPM or RCC^29^. Furthermore, we also demonstrated that hematological cancers such as T-anaplastic large cell lymphoma^30,31^ and multiple myeloma^32^ are also potential targets of CD26-directed therapies as well as MPM and RCC.

Therefore, herein we initially investigated the therapeutic efficacy of DPP4 inhibitors on multiple myeloma cells, work which subsequently led to the interesting findings indicating that DPP8 is a novel therapeutic target for multiple myeloma.

## Results

### Cytotoxic effects of DPP4 inhibitors against multiple myeloma cell lines

**Figure 1:**
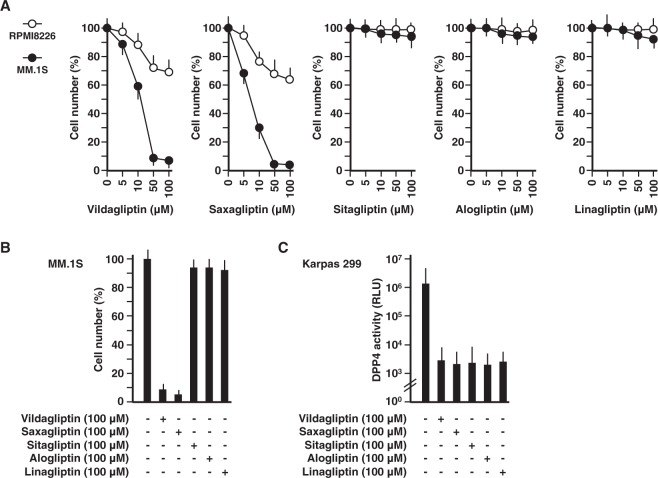
Cytotoxic effects of DPP4 inhibitors against multiple myeloma cell lines. (**A**) 1.0 × 10^5^ MM.1 S (open circles) or RPMI8226 (closed circles) cells were cultured at doses of 0–100 µM DPP4 inhibitors (vildagliptin, saxagliptin, sitagliptin, alogliptin, or linagliptin) for 72 hours. Cell number was estimated by a colorimetric assay using WST-1 reagent (n = 6). (**B**) 1.0 × 10^5^ MM.1 S cells were cultured with 100 µM DPP4 inhibitors (vildagliptin, saxagliptin, sitagliptin, alogliptin, or linagliptin) for 72 hours. Cell number was estimated by a colorimetric assay using WST-1 reagent (n = 6). (**C**) 1.0 × 10^5^ Karpas 299 cells were cultured with 100 µM DPP4 inhibitors (vildagliptin, saxagliptin, sitagliptin, alogliptin, or linagliptin) for 24 hours, respectively. DPP4 activity was estimated using a luminogenic DPP4 substrate, Gly-Pro-aminoluciferin (n = 6). The data are representative of three separate experiments and presented as the mean ± SD.

The cytotoxic effects of DPP4 inhibitors on multiple myeloma cell lines were examined using WST-1 cell proliferation assay system as shown in Fig. 1A. Vildagliptin treatment up to 100 µM led to decreased cell number of MM.1 S or RPMI8226 cells in a concentration-dependent manner till 7 and 70%, respectively. Nevertheless, 100 µM of vildagliptin is not achieved as a plasma concentration by the recommended oral daily dose (i.e. 100 mg) since oral administration of 200 mg of vildagliptin resulted in less than 5.0 µM of plasma concentration as demonstrated previously^33^. Similar cytotoxic effects were observed when cells were treated with saxagliptin; however, both cell lines were unaffected in the presence of sitagliptin, alogliptin, or linagliptin. As only vidagliptin and saxagliptin showed the marked cytotoxicity (Fig. 1B), it was assumed that the cytotoxicity of those two DPP4 inhibitors was due to stronger suppressive effects on DPP4 activity than the other three DPP4 inhibitors. However, surprisingly, the suppressive effects of these five DPP4 inhibitors on DPP4 activity were almost identical (Fig. 1C). In addition, the cytotoxic effects against the T-cell lymphoma cell line Karpas 299 was also observed only with vildagliptin and saxagliptin. (Supplementary Fig. 1A). These results indicated that vildagliptin and saxagliptin exerted their anti-myeloma activity by other mechanisms than DPP4-inhibition.

### Anti-myeloma activity of DPP8/9 inhibitor

**Figure 2:**
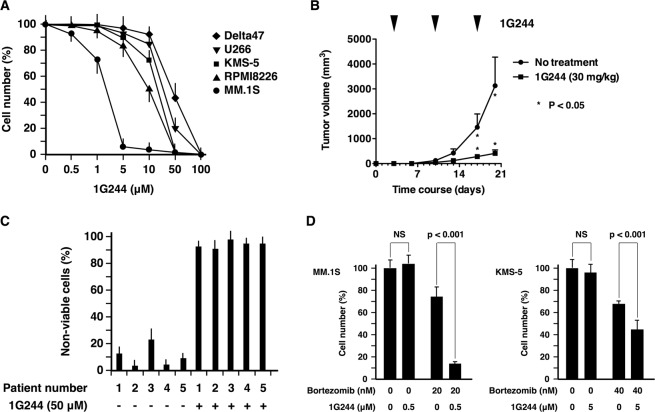
Anti-myeloma activity of DPP8/9 inhibitor. (**A**) 1.0 × 10^5^ Delta47 (rhombuses), U266 (inverted triangles), KMS-5 (squares), RPMI8226 (triangles), or MM.1 S (circles) cells were cultured with 1G244 (0–100 µM) for 72 hours. Cell number was estimated by a colorimetric assay using WST-1 reagent (n = 6). (**B**) 0.5 × 10^5^ MM.1 S cells were subcutaneously inoculated into NOG mice (n = 6). Three days after the inoculation, 1G244 (30 mg/kg) was administered subcutaneously once-a-week. The tumor volume was measured every third or fourth day. (**C**) 0.1–0.5 × 10^5^ CD138 + myeloma cells from patients were cultured with 1G244 (50 µM) for 24 hr (patient number 2–5) or 48 hr (patient number 1) Non-viable cells were estimated by a flow cytometric analysis using 7-AAD reagent (n = 3). (**D**) 1.0 × 10^5^ MM.1 S (left panel) or KMS-5 (right panel) cells were cultured with bortezomib (20 or 40 nM) with or without 1G244 (0.5 or 5 µM) for 72 hours, respectively. Cell number was estimated by a colorimetric assay using WST-1 reagent (n = 6). The data are presented as the mean ± SD.

Based on previous work showing that vildagliptin and saxagliptin were classified into the same category (Class 1) of DPP4 inhibitors^34^ and had non-negligible off-target effects on DPP8/9 activity^35^, we hypothesized that vildagliptin and saxagliptin-induced inhibitory effects on DPP8/9 were the causal factor for their anti-myeloma activity. To further address this topic, we employed a specific DPP8/9 inhibitor, 1G244^36^ to confirm whether DPP8/9 inhibition actually induced cell death in multiple myeloma cells. As shown in Fig. 2A, 1G244 dose-dependently decreased viable cell number of five multiple myeloma cell lines as well as three T-cell lymphoma cell lines (Supplementary Fig. 1B). Almost complete cell death of all cell lines was observed at a dose of 100 µM. However, since it is known that 100 µM of 1G244 induced nonspecific cell death^37^; we therefore used 1G244 at levels below 50 µM in our subsequent experiments. Since, MM.1 S was the most susceptible among five cell lines, it was inoculated into mice to confirm the anti-myeloma effects of 1G244 *in vivo*. 1G244 was administered subcutaneously into mice at 30 mg/kg once a week, since daily intravenous injection of 1G244 at the same dosage has been linked to severe cyanosis in rats on day 4 or 5^36^. Once-a-week administration of 1G244 apparently suppressed the subcutaneous growth of MM.1 S cells with no other obvious clinical symptoms in mice (Fig. 2B). 1G244 effect on samples from patients with multiple myeloma was also examined. CD138-positive myeloma cells were efficiently isolated from bone marrow cells using magnetic beads (Supplementary Fig. 2). Myeloma cells of all five patients were regarded as non-viable (91–97%) following incubation with 50 µM 1G244 (Fig. 2C). It should be noted that myeloma cells of patient 2 had a deletion of chromosome 17p, being resistant to various chemotherapeutic and biologic agents such as bortezomib, lenalidomide, dexamethasone, cyclophosphamide, and doxorubicin. These results suggested that DPP8/9 inhibition induced cell death in myeloma cells via a distinctive signaling pathway which did not overlap with that of existing chemotherapeutic and biologic agents. Therefore, the effect of the combination of 1G244 and bortezomib was examined (Fig. 2D). 1G244 at a dose of 0.5 µM displayed no cytotoxic effects on MM.1 S cells; however, in combination with bortezomib (20 nM), the number of viable cells decreased significantly compared to the effect of bortezomib (20 nM) alone. Similar effects were observed on another myeloma cell line, KMS-5. These results suggested that DPP8/9 is a realistic potential therapeutic target for the management of multiple myeloma; however, it was reported that the cell death of THP-1 monocytes induced by 1G244 was not dependent on DPP8/9^38^. Therefore, we introduced small interfering RNAs (siRNAs) into our experiments to downregulate specifically the expression of either DPP8 or DPP9.

### DPP8 as a target of myeloma therapy

**Figure 3:**
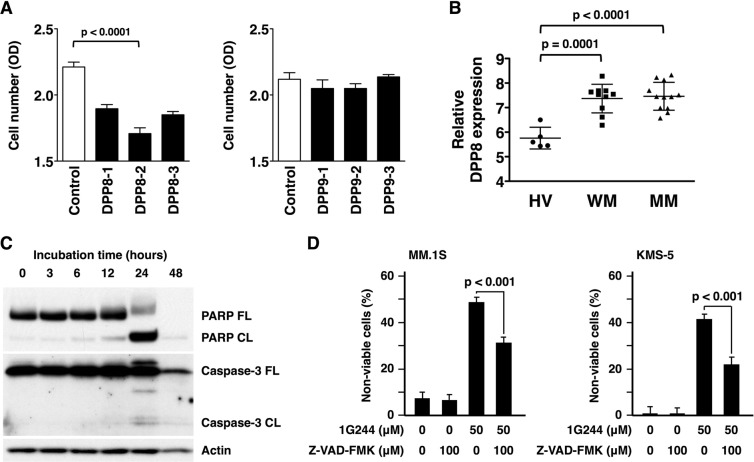
DPP8 as a target of myeloma therapy. (**A**) 1.0 × 10^5^ MM.1 S cells were cultured with 20 nM DPP8 siRNAs (left) or DPP9 siRNAs (right) for 72 hours. Cell number was estimated by a colorimetric assay using WST-1 reagent (n = 6). (**B**) DPP8 gene expression of CD38 + bone marrow cells in healthy volunteers (HV) (n = 5) was compared to those in Waldenstrom’s macroglobulinemia patients (WM) (n = 10) or in multiple myeloma (MM) patients (n = 12) based on a dataset record GDS2643. (**C**) 1 × 10^6^ MM.1 S cells were cultured with 1G244 (50 µM) for 0–48 hours. The full length (FL) and cleaved form (CL) of PARP or caspase-3 were detected by Western blot analysis. β-Actin was used as a loading control. Full-length blots are presented in Supplementary Fig. 3. (**D**) 1.0 × 10^6^ MM.1 S (left) or KMS-5 (right) cells were cultured with 1G244 (50 µM) with or without pan-caspase inhibitor, Z-VAD-FMK (100 µM) for 24 hours. Non-viable cells were estimated by a flow cytometric analysis using 7-AAD reagent (n = 6). The data are representative of three separate experiments except (**B**) and presented as the mean ± SD in (**A**,**B**,**D**).

In order to determine whether DPP8 or DPP9 is particularly responsible for the anti-myeloma activity of 1G244, we employed small interfering RNAs (siRNAs) specific to either DPP8 or DPP9, due to unavailability of specific chemical inhibitors to either DPP8 or DPP9 since these two DPPs closely resemble each other in structure^39,40^. As shown in Fig. 3A, treatment with siRNAs specific to DPP8 clearly decreased viable cell number; however, treatment with siRNA specific to DPP9 showed no such change. These results indicated that DDP8 is a novel target for myeloma therapy. Indeed, DPP8 was expressed at a higher level in CD38+ bone marrow cells of Waldenstrom’s macroglobulinemia and multiple myeloma patients than those of healthy volunteers (Fig. 3B). Regarding the potential cellular mechanism involved in 1G244-induced cell death in multiple myeloma cells, apoptosis was the most likely, as cleaved forms of both caspase-3 and PARP were detected (Fig. 3C). Furthermore, 1G244-induced cell death was suppressed by the pan-caspase inhibitor Z-VAD-FMK (Fig. 3D). These findings led to our conclusion that the mechanism for cell death of multiple myeloma cells induced by DPP8-inhibition is apoptosis.

## Discussion

Recently, inhibition of DPP8/9 has garnered attention as a new potential therapeutic strategy for acute myeloid leukemia (AML). Johnson DC *et al*. reported that Val-boroPro, a non-selective inhibitor of the post-proline cleaving serine proteases, as well as the specific DPP8/9 inbibitors 1G244 and L-allo-Ile-isoindoline induced cell death in many AML cell lines and primary AML samples^37^. Although it was demonstrated that Val-boroPro had no activity against any of the non-AML cell lines, it did not necessarily contradict our findings that DPP8/9 inhibition induced cell death in multiple myeloma cells. This is due to the fact that the twenty non-AML cell lines which were tested did not include multiple myeloma cell lines. Therefore, our present paper reports for the first time the anti-myeloma activity induced by the DPP8/9 inhibitor.

Meanwhile, Johnson DC *et al*. concluded that pyroptosis, an immunostimulatory form of programmed cell death, was the mechanism responsible for cell death induced by the DPP8/9 inhibition, being dependent on caspase-1 activation downstream of inflammasome formation^37^. In their report, caspase-1 expression was found as a key determinant of cell sensitivity to a DPP8/9 inhibitor and that treatment of sensitive cells with a DPP8/9 inhibitor induced the cleavage of pyroptotic substrate gasdermin D (GSDMD) but not the apoptotic substrate PARP. However, in contrast, our data showed that caspase-3 and PARP cleavage was clearly detected in 1G244-mediated cell death of multiple myeloma cells (Fig. 3C). We thus concluded that apoptotic cell death signaling was induced in multiple myeloma cells by DPP8/9 inhibition. Our conclusion does not completely oppose the possible involvement of pyroptotic cell death because cell death signaling involves a complex process. The inflammasome formation can activate caspase-8, which mediates the activation of downstream caspases such as caspase-3, caspase-7, and caspase-9^41–43^. Besides, caspase-3 can be activated downstream of caspase-1 through inflammasome responses independent of caspase-8^44^. In the case of 1G244-dependent caspase-3 cleavage, additional detailed work is required to examine the potential involvement of inflammasome responses.

During the investigation of DPP8/9 inhibition-induced pyroptotic cell death signaling of AML cells, caspase recruitment domain-containing protein 8 (CARD8) was identified as an activator of pro-caspase-1^37^. Concerning the mechanism involved in DPP8/9 inhibition-mediated activation of CARD8, DPP9 was regarded as a novel interacting partner with CARD 8 as well as the NLR family member, pyrin domain containing 1 (NLRP1)^45^. NLRP1 is the human homolog of the mouse Nlrp1b, which is activated in murine macrophage cells treated with the DPP8/9 inhibitors^46^. DPP9 functions as an endogenous inhibitor of NLRP1; therefore, DPP8/9 inhibition activates NLRP1, leading to pyroptotic cell death. Interestingly, DPP9 contributes to the inhibition of NLRP1 not only by its catalytic function but also by physical interaction with NLRP1. These findings are based on the data showing that the catalytically inactive DPP9 point mutant S759A, which does not compromise the ability of DPP9 to bind NLRP1, led to significant but partial repression of NLRP1-dependent pyroptosis reaction, and DPP8/9 inhibitors caused the dissociation of DPP9 from NLRP1. While the inhibitory mechanism of DPP9 on NLRP1-dependent pyroptosis by its physical interaction with NLRP1 needs to be investigated further, it is important to note that some other biological function of DPP9 other than its catalytic activity may be revealed in the future.

While either CARD8 or NLRP1 may be involved in DPP9-dependent pyroptosis, a mechanism involved in DPP8-induced apoptotic cell death has not yet been elucidated. The catalytic activity of DPP8 is the most probable function that contributes to the induction of apoptotic cell death signaling; however, other possible involvement of its non-catalytic function such as protein interaction may also play a role.

In the present study, we speculate that its inhibitory effect on DPP8/9 is responsible for the anti-myeloma activity of vildagliptin; however, “off-target” effects should be taken into consideration when vildagliptin is used as an anti-cancer drug. Regarding this point, recent investigation demonstrated that vildagliptin reduced lung cancer growth exerted by surfactant-activated macrophages and NK cells via tumor necrosis-related apoptosis-inducing ligand (TRAIL)-mediated cytotoxicity^22^.

In summary, our present work demonstrated that DPP8 is a novel target for multiple myeloma therapy inducing apoptotic cell death. Further development of specific inhibitors against DPP8 would provide promising therapeutic effects in human multiple myeloma.

## Methods

### Cell culture

Five human multiple myeloma cell lines, Delta47, U266, KMS-5, RPMI8226, and MM.1 S cells as well as three human T-cell lymphoma cell lines, Karpas 299, H9, and HUT102 cells were supplied by American Type Culture Collection (Manassas, VA) and maintained in RPMI 1640 (Gibco BRL, Tokyo, Japan) with 10% heat-inactivated fetal bovine serum (Sigma, St. Louis, MO).

### Reagents

Benzyloxycarbonyl-Val-Ala-Asp (OMe) fluoromethylketone (Z-VAD-FMK) was purchased from Medical and Biological Laboratories (Nagoya, Japan). 1G244 was purchased from AK Scientific (Union city, CA). Alogliptin was purchased from ChemScene (Monmouth Junction, NJ). Linagliptin was purchased from BioVision (Milpitas, CA). Sitagliptin and Bortezomib were purchased from Santa Cruz Biotechnology (Santa Cruz, CA). Vildagliptin was purchased from LKT Laboratories (St. Paul, MN). Saxagliptin was purchased from Adooq Bioscience (Irvine, CA).

### Cellular cytotoxicity

The number of viable cells seeded onto a 96-well culture plate was quantified using Premix WST-1 Cell Proliferation Assay System (TaKaRa, Kyoto, Japan) according to the manufacturer’s instructions. Briefly, 10 μl of Premix WST-1 per 100 μl of culture medium was added to each well and the cells were incubated under the standard culture condition for 1 hour. WST reduction was determined with an automated ELISA plate reader, ImmunonMini NJ-2300 spectrophotometer (InterMed, Tokyo, Japan), at an optical density (OD) of 450–650 nm, as we described previously^47^.

### DPP4 activity

The DPP4 activity of the cell culture media was measured using DPPIV-Glo^TM^ Protease Assay Kit (Promega, Madison, WI) according to the manufacturer’s instructions. Briefly, 5 μl of the cell culture media was added to the mixture of a luminogenic substrate, Gly-Pro-aminoluciferin and a recombinant luciferase. After the release of aminoluciferin, substrate for luciferase by DPP4 cleavage and the following luciferase reaction, luminescence was recorded as relative light units (RLU) on a plate reader, Infinite M1000 Pro (Tecan, Männedorf, Switzerland).

### *In vivo* studies

NOD/Shi-scid IL-2Rγnull (NOG) female mice of age (6–7 weeks) and weight (19–21 g) were obtained from Central Institute for Experimental Animals (CIEA) (Kawasaki, Japan). The mice were kept under specific pathogen-free conditions with a 12 hour day and night cycle with free access to food and water, and received humane care in compliance with Institutional Guidelines. All experiments were approved by the Animal Care and Use Committee of Sapporo Medical University and were performed in accordance with the guidelines and regulations of the Animal Care and Use Committee of Sapporo Medical University. In order to examine the anti-myeloma activity of 1G244, 5 × 10^6^ MM.1 S cells were inoculated subcutaneously on the left side at the back of NOG mice. Three days after the inoculation, 30 mg/kg of 1G244 was administered subcutaneously once-a-week. The growth of tumor was followed every third or fourth day by measurements with a caliper and its volume was calculated according to the following formula: MD × TL^2^ × 1/2, where MD and TL are the maximum diameter and transverse length, respectively. The mice were sacrificed before the volume of the tumor mass reached 3,500 mm^3^ for ethical reason, as we described previously^30^.

### Myeloma cells from patients

Multiple myeloma patients followed up between January 2000 and December 2015 in our hospital were retrospectively screened. Frozen bone marrow cells from five patients were obtained for analysis. Myeloma cells were positively selected using MACSprep^TM^ Multiple Myeloma CD138 MicroBeads, human Kit (Miltenyi Biotec, Auburn, CA) according to the manufacturer’s instructions. Briefly, bone marrow cells were suspended in MACS buffer and incubated with microbeads conjugated to monoclonal anti-human CD138 antibodies. The cells were then loaded onto MACS column. The magnetic labeled CD138+ cells were bound to the column and released from magnetic field using wash buffer. All patients were treated according to institutional review board-approved protocols and gave informed consent in accordance with the Declaration of Helsinki. This study was approved by the institutional review board of Sapporo Medical University.

### Flow cytometry

The population of non-viable cells was estimated by a flow cytometric analysis using a standard flow cytometric viability probe, 7-Amino-Actinomycin (7-AAD) reagent (BD Biosciences, San Jose, CA) which permeates the membranes of both dead and damaged cells. Briefly, after the incubation with 7-AAD for 15 minutes at room temperature in the dark, cells were analyzed on the BD FACSCanto II (BD Biosciences) with FlowJo software 7.6.1 (Treestar, Ashland, OR).

### Gene expression database

A dataset record GDS2643 was found in the Gene Expression Omnibus (GEO) database at the National Center for Biotechnology Information (NCBI) (http://www.ncbi.nlm.nih.gov/geo/) and used to compare DPP8 gene expression of CD38+bone marrow cells in healthy volunteers (HV) to those in Waldenstrom’s macroglobulinemia patients (WM) or in multiple myeloma (MM) patients.

### Preparation of siRNAs

Stealth siRNAs (Set of 3)^TM^ (Invitrogen, Carlsbad, CA) targeting human DPP8 with the following sequences were used: HSS123433 (DPP8-1) (gga agg auc aua gau guc aua gau a); HSS123434 (DPP8-2) (gga ccu cau uca gac aga auc uau u); HSS123435 (DPP8-3): gcc ggu agu gga auu uau cac gua a. As a negative control, Low GC in Stealth RNAi Negative Control Kit^TM^ (Invitrogen) was used. Also, Stealth siRNAs (Set of 3)^TM^ targeting human DPP9 with the following sequences were used: HSS132085 (DPP9-1) (gac agg cag caa gaa ucc caa gau u); HSS132086 (DPP9-2) (gca agu acu cgg gcc uca uug uca a); HSS132087 (DPP9-3) (ccu gga agc aga ugc ugg auc auu u). As a negative control, Medium GC in Stealth RNAi Negative Control Kit^TM^ was used.

### Transfection of siRNAs

Multiple myeloma cells were transfected with siRNAs using Lipofectamine RNAiMAX Reagent (Invitrogen) according to the manufacturer’s instructions. Briefly, MM.1 S cells were seeded at 1.0 × 10^5^ cells/100 µL/well onto 96-well plates. The cells were then transfected with 2 pmol siRNA and 0.3 µL Lipofectamine RNAiMAX Reagent diluted with Opti‐MEM Medium (Invitrogen) and cultured for 72 hours. This gives a final siRNA concentration of 20 nM.

### Western blot analyses

Cells were lysed in a buffer containing 1% sodium dodecyl sulfate (SDS), 20 mM Tris-HCl pH 7.4, 5 μg/ml pepstatin A, 10 μg/ml leupeptin, 5 μg/ml aprotinin and 1 mM phenyl-methylsulfonyl fluoride and then heated for 5 minutes. After passage through a 20-gauge needle ten times and centrifugation at 15,000 rpm at 4 °C for 30 minutes, the aliquot was boiled in a standard reducing sample buffer for 3 minutes and subjected to SDS-polyacrylamide gel electrophoresis. It was followed by transfer to Immobilon-P membrane (Millipore, Bedford, MA) and hybridization with anti-poly (ADP-ribose) polymerase (PARP) antibody (#9542) (Cell Signaling, Danvers, MA), anti-caspsae-3 antibody (#9662) (Cell Signaling), and anti-actin antibody (sc-1615) (Santa Cruz Biotechnology). Proteins detected by these antibodies were visualized with horseradish peroxidase–conjugated anti-rabbit or goat antibody (Santa Cruz Biotechnology) followed by the use of enhanced chemiluminescence (Amersham Pharmacia Biotech, Uppsala, Sweden), as we described previously^47^.

### Statistical analysisn

The statistical significance of difference was evaluated by Student’s t-test using GraphPad Prism version 5.0 (GraphPad Software, La Jolla, CA). Statistical significance of p < 0.05 considered significant.

## Supplementary information

Supplementary information
