# Supplementary material for: DPP8 is a novel therapeutic target for multiple myeloma
Source: Sci Rep. 2019 Dec 2;9:18094. doi: 10.1038/s41598-019-54695-w (PMC6889119; doi:10.1038/s41598-019-54695-w)
Supplement: Supplementary file 1 — Supplementary information [file 41598_2019_54695_MOESM1_ESM.pdf]

Title

DPP8 is a novel therapeutic target for multiple myeloma

Authors and affiliations

Tsutomu Sato[1], Ayumi Tatekoshi[2], Kohichi Takada[2], Satoshi Iyama[2], Yusuke Kamihara[3], Paras Jawaid[4], Mati Ur Rehman[4], Kyo Noguchi[4], Takashi Kondo[4], Sayaka Kajikawa[1], Kotaro Arita[1], Akinori Wada[1], Jun Murakami[1], Miho Arai[5], Ichiro Yasuda[6], Nam H. Dang[7], Ryo Hatano[8], Noriaki Iwao[8], Kei Ohnuma[8], and Chikao Morimoto[8]

[1]Department of Hematology, Toyama University Hospital, Toyama, Japan
[2]Department of Medical Oncology and Hematology, Sapporo Medical University, Sapporo, Japan
[3]Department of Hematology, National Cancer Center Hospital East, Kashiwa, Japan
[4]Department of Radiology, University of Toyama, Toyama, Japan
[5]Department of Pediatrics, University of Toyama, Toyama, Japan
[6]Department of Gastroenterology and Hematology, University of Toyama, Toyama, Japan
[7]Division of Hematology/Oncology, University of Florida, Gainesville, Florida, USA
[8]Department of Therapy Development and Innovation for Immune Disorders and Cancers, Juntendo University, Tokyo, Japan

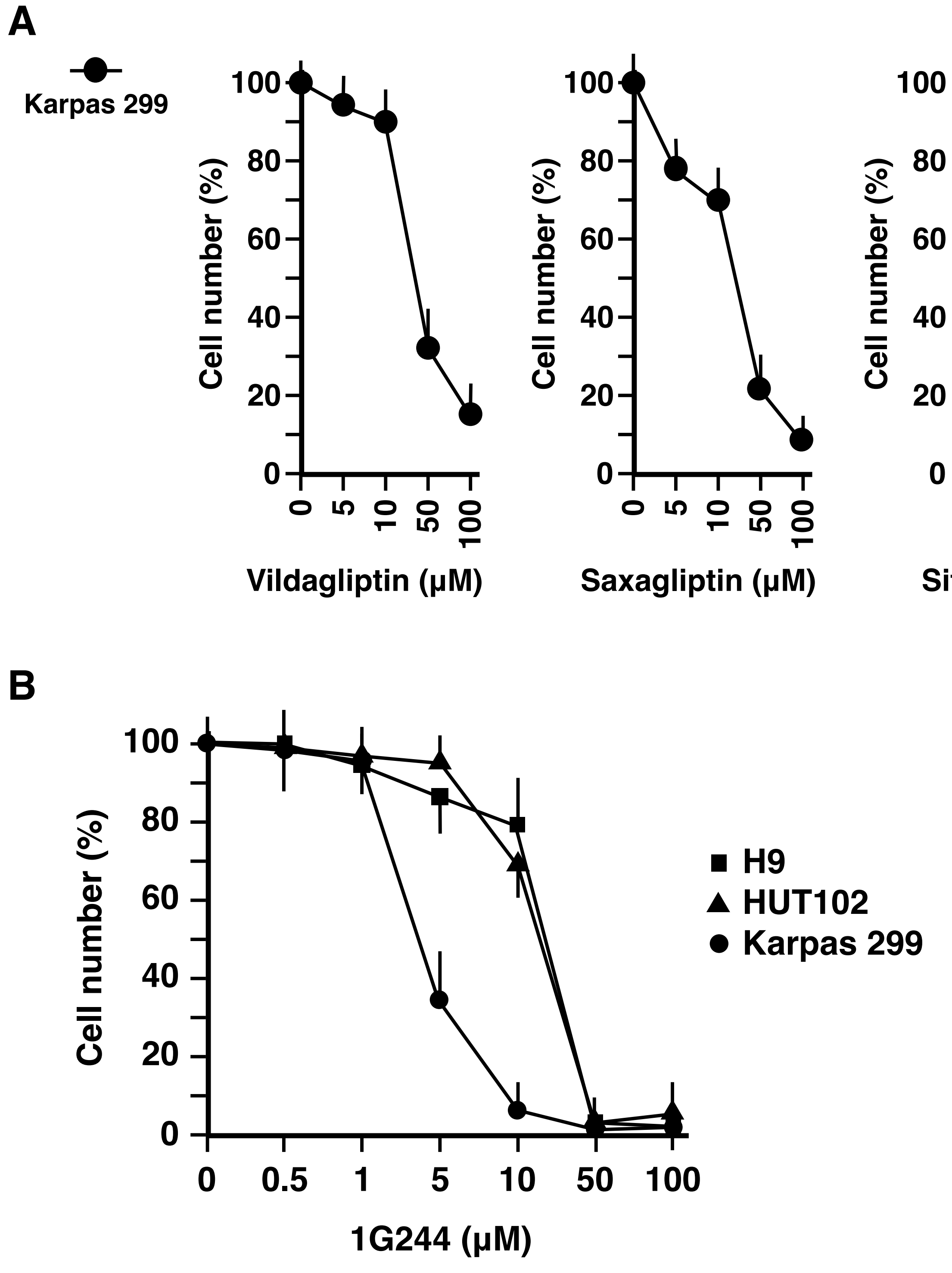

Supplementary Fig. 1

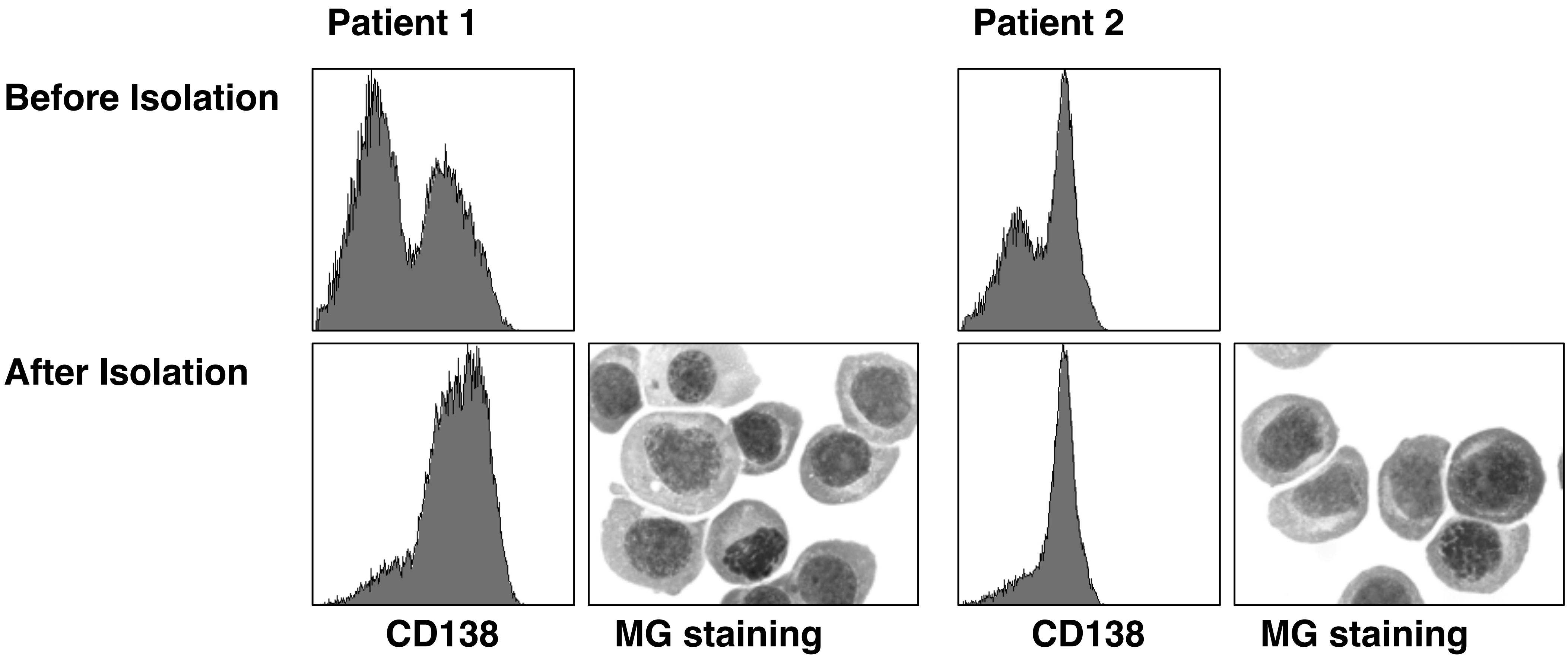

Supplementary Fig. 2

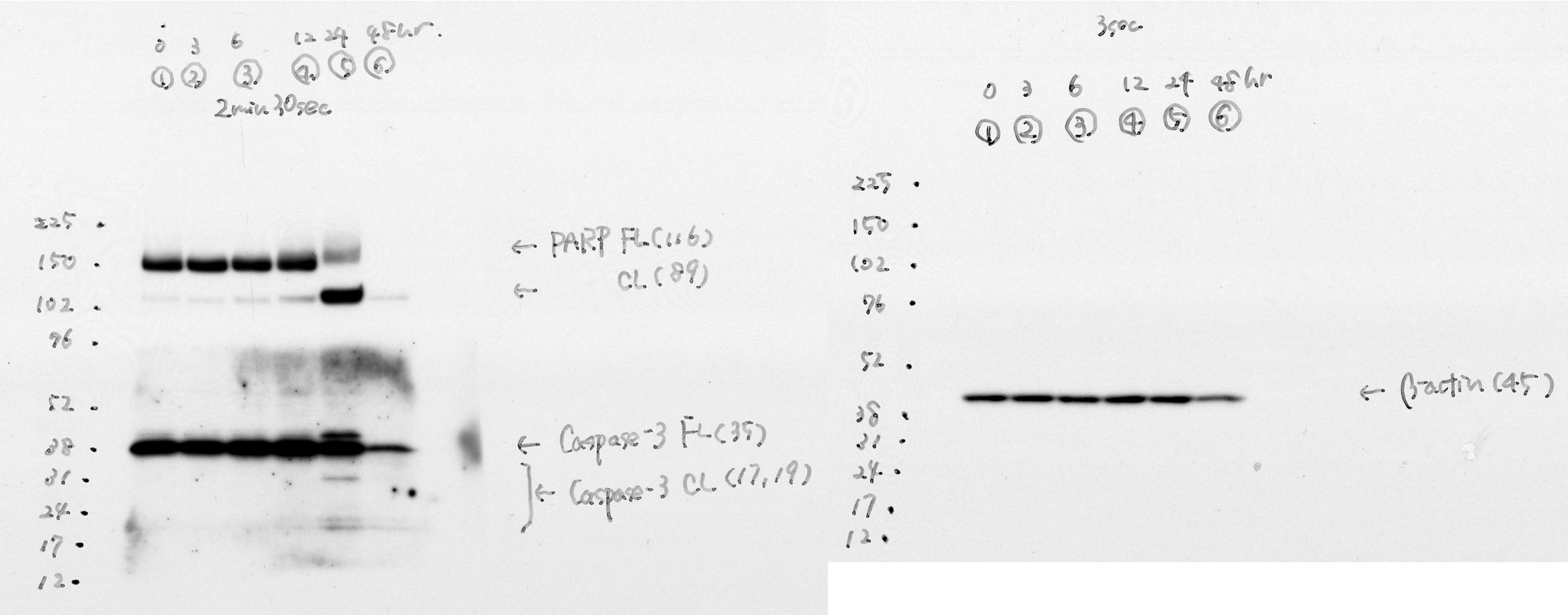

Supplementary Fig. 3

Supplementary Figure Legend

Supplementary Fig. 1 Cytotoxic effects of DPP8/9 inhibitor against T-cell lymphoma cell lines

(A) 1.0×10^5 Karpas 299 (open circles) cells were cultured with 0-100 μM DPP4 inhibitors (vildagliptin, saxagliptin, sitagliptin, alogliptin, or linagliptin) for 72 hours. Cell number was estimated by a colorimetric assay using WST-1 reagent (n=6).

(B) 1.0×10^5 H9 (squares), HUT102 (triangles), or Karpas 299 (circles) cells were cultured with 1G244 (0-100 μM) for 72 hours. Cell number was estimated by a colorimetric assay using WST-1 reagent (n=6).

Supplementary Fig. 2 Myeloma cells from patients

CD138-positive myeloma cells were isolated from bone marrow cells using magnetic beads. The expression of CD138 was analyzed flow cytometricaly before and after isolation. The isolated cells were stained with May-Giemsa method. As the typical example, the data of patient 1 and 2 are presented.

Supplementary Fig. 3 Original uncropped full-length images of the western blots shown in Fig. 3C
